# Iodothyronine Interactions with the System L1 Amino Acid Exchanger in 3T3-L1 Adipocytes

**DOI:** 10.4061/2010/726098

**Published:** 2010-06-24

**Authors:** Fiona E. Mitchell, Lisa A. Roy, Peter M. Taylor

**Affiliations:** Division of Molecular Physiology, College of Life Sciences, James Black Centre, University of Dundee, Dundee DD1 5EH, UK

## Abstract

Thyroid hormones enter isolated white adipocytes largely by a System L1-type amino acid transporter *en route* to exerting genomic actions. Differentiated 3T3-L1 mouse adipocytes in culture express mRNA for LAT1 (the catalytic subunit of high-affinity System L1). L-[^125^I]-T_3_ uptake into 3T3-L1 adipocytes included a substantial saturable component inhibited by leucine. L-[^3^H]phenylalanine uptake into 3T3-L1 cells was saturable (*K*_*m*_ of 31 *μ*M), competitively inhibited by T_3_ (*K*_*i*_ of 1.2 *μ*M) and blocked by leucine, BCH, and rT_3_ as expected for substrate interactions of System L1. Efflux of preloaded L-[^3^H]phenylalanine from 3T3-L1 adipocytes was *trans* stimulated by external leucine, demonstrating the obligatory exchange mechanism of System L1 transport. T_3_ (10 *μ*M) did not significantly *trans* stimulate L-[^3^H]phenylalanine efflux, but did competitively inhibit the *trans* stimulatory effect of 10 *μ*M leucine. The results highlight strong competitive interactions between iodothyronines (T_3_, rT_3_) and amino acids for transport by System L1 in adipocytes, which may impact cellular iodothyronine exchanges during altered states of protein nutrition.

## 1. Introduction

The thyroid hormones (THs) L-thyroxine and L-triiodothyronine are iodothyronines exerting their major physiological effects by regulation of gene expression in target cells, which they must first enter by crossing the plasma membrane [1–3]. Iodothyronines are now known to translocate cell membranes by a variety of mechanisms, which include the sharing of transport systems for large neutral amino acids (LNAAs; principally aromatic and branched-chain amino acids) and organic anions (see, e.g., [4–6] for review). TH retain a tyrosine-derived amino acid moiety within the iodothyronine molecular structure, allowing them to be accepted as substrates by LNAA transporters such as System L (notably the “System L1” SLC7A5/SLC3A2 heterodimer isoform LAT1) [7, 8] and System T (MCT10; SLC16A10) [9].

White adipose tissue is an important target tissue for TH action, where effects include stimulation of adipogenesis itself [10], modulation of fatty acid synthesis via regulation of expression of lipogenic enzymes [10–12] and modulation of leptin secretion [13, 14]. TH enter rat adipocytes largely by a System L1-type amino acid transporter [15] and LAT1 mRNA are expressed in rat adipocytes [16]. System L1 is also likely to be of major importance for amino acid metabolism in adipose tissue, which is a site of significant BCAA degradation and utilization for fatty acid and sterol synthesis [17, 18]. The System L1 substrate leucine in particular appears to be involved in modulation of the mTOR signalling pathway in adipocytes [19]. The activity and expression of System L1 in adipocytes, and its regulation by TH, may therefore be of quantitative importance for whole body iodothyronine and amino acid turnover. In the present study using differentiated 3T3-L1 mouse adipocytes [20, 21], we investigate how the mechanism of System L1 transport is influenced by iodothyronines. 

## 2. Experimental Procedures

### 2.1. Materials

L-[2,3,4,5,6-^3^H]Phenylalanine was obtained from Amersham (GE Healthcare, Slough, U.K.) and L-[^125^I]-Triiodothyronine from PerkinElmer (Bucks, UK). Cell culture media were obtained from GibcoBRL Life Technologies (Paisley, UK) unless stated otherwise; other chemicals were obtained from either Sigma Chemicals (Poole, Dorset, UK) or BDH Merck Ltd (Poole, Dorset, UK).

### 2.2. Cell Culture

3T3-L1 mouse fibroblasts (ATCC CL-173) were cultured at 37°C, 95% air: 5% CO_2_ in Dulbecco's Modified Eagles Medium (DMEM; high-glucose) with 10% Donor Bovine Serum (DBS) and 1% antibiotic/antimycotic (A/A) solution. Before reaching confluence, cells were resuspended by trypsinisation and reseeded into 6- or 12-well experimental plates. To differentiate into adipocytes [22], the 3T3-L1 fibroblasts were grown to confluence (preadipocytes) and then the medium was changed to DMEM with 10% foetal bovine serum (FBS) and 1% A/A solution over the duration of the differentiation process. For the first two days of differentiation, 1 *μ*g·mL^−1^ insulin, 5 *μ*M isobutylmethylxanthine, and 100 pM dexamethasone were also added to the media and for the subsequent two days 1 *μ*g·mL^−1^ insulin alone was added. The adherent cells were allowed to fully differentiate over a further six days, then used for experiments on days 11 or 12.

### 2.3. [^125^I]-T_3_ and [^3^H]Phenylalanine Uptakes

Immediately prior to an experiment, media was aspirated from the wells and the cells were washed twice in PBS. Transport buffer (121 mM NaCl, 4.9 mM KCl, 2.5 mM MgSO_4_, 20 mM Tris-HCl, 1 mM CaCl_2_, pH 7.4) containing either L-[^125^I]-T_3_ at 30 kBq·mL^−1^ or L-[^3^H]phenylalanine at 18.5 kBq·mL^−1^ and any other necessary compounds (e.g., inhibitors) were then added to the cells. Once the cells had been incubated in the transport buffer for the designated time period, the buffer was aspirated and the cells were washed quickly three times in cold PBS to halt the uptake process. The cells were then lysed in 1.25 mL 50 mM NaOH overnight at room temperature prior to assay of radioactivity by liquid scintillation counting and determination of protein concentration using Bradford reagent (Biorad UK). Preliminary experiments (data not shown) demonstrated that the uptake of 5 *μ*M [^3^H]phenylalanine was linear for at least 5 minutes and therefore, in all subsequent experiments reported here, phenylalanine uptake was measured over a 3-minute period. L-[^125^I]-T_3_ uptakes were measured over a 10-minute period [15]. The specific activity (DPM·pmol^−1^) of radiotracers was calculated from the radioactivity (DPM) of 10 *μ*L transport buffer measured using liquid scintillation counting; tracer uptake is expressed as pmol·mg protein^−1^·10 min. For *trans* stimulation studies, 3T3-L1 adipocytes were preincubated in transport buffer containing 2 mM leucine for 15 min prior to the initial PBS washes.

### 2.4. [^3^H]Phenylalanine Efflux

3T3-L1 cells were preloaded with [^3^H]phenylalanine for a period of 15 minutes according to the uptake protocol described above. The radioactive buffer was then aspirated off and the cells were rapidly washed three times with PBS. The cells were then re-incubated in (nonradioactive) transport buffer, half of which was removed at timed intervals and immediately replenished with fresh buffer. At the end of each experiment, the remaining buffer was aspirated and the cells lysed overnight in 1.25 mL 50 mM NaOH. The incubation buffer aliquots and cell lysates were assayed for radioactivity and the lysate also for protein concentration as described above.

### 2.5. RNA Extraction and RT-PCR

RNA was extracted from cells using TRIzol Reagent (Invitrogen, Poole, UK) according to the manufacturer's instructions, resuspended in RNase-free H_2_O and quantified by UV spectrometry. 1 *μ*g RNA was denatured in the presence of 0.5 *μ*g OligodT (Oligo Synthesis Service, University of Dundee) at 70°C for 5 min then reverse-transcribed using Moloney Murine Leukemia Virus Reverse Transcriptase (M-MLV RT) at 100 U/*μ*g RNA in 25 *μ*L reaction buffer including 500 *μ*M dNTPs (Fermentas) at 42°C for 1 hour. The resulting first-strand cDNA was stored at −20°C prior to use in PCR using gene-specific primers to test for specific mRNA expression. The primer sets used in the PCR program were

 LAT1 forward 5′-tctccttgcccattgtcacc-3′
     reverse    5′-atgactcccaggtggtagttcc-3′
 LAT2 forward 5′-aagggaacccgacagcgaaacaac-3′
     reverse    5′-gggggaagcaggtagggaagagtg-3′.

For each PCR, 2 *μ*L of first strand DNA, 1 *μ*M each primer and standard 2x GoTaq Green PCR Master Mix (Promega) were used in a 20 *μ*L total volume. The PCR programme used was 95°C, 3 min; [94°C, 30 s; 55°C, 30 s; 72°C, 1 min] 40 cycles; 72°C, 2 min using a Thermo Scientific Hybaid Px2 thermal cycler.

### 2.6. Data Analysis

The data are expressed as means +/− S.E.M for *n* adipocyte preparations. Statistical significance for uptake measurements was assessed by one-way ANOVA followed by a Dunnet *post hoc* test. For efflux measurements, Graphpad software (San Diego, CA, USA) was used to determine whether the gradients of the lines of best fit (rate constants) were significantly different from control. Differences were considered significant where *P* < .05.

## 3. Results

### 3.1. L-T_3_ Uptake

**Figure 1:**
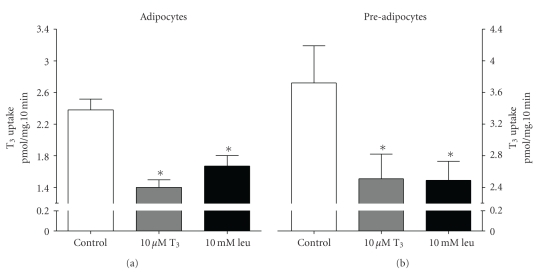
Uptake of the iodothyronine L-T_3_ into 3T3-L1 adipocytes and pre-adipocytes. [^125^I]-T_3_ uptake was measured at 50 nM over 10 minutes in the absence (control) or presence of 10 *μ*M unlabelled T_3_ or 10 mM leucine in the uptake buffer. Values are expressed ± SEM of 6–12 measurements. *P < .05; value significantly different from control using Dunnett's Multiple Comparison Test.

The uptake of 50 nM L-[^125^I]-T_3_ into 3T3-L1 adipocytes included a substantial saturable component, being reduced by around 40% on addition of 10 uM unlabelled T_3_ (Figure 1). The magnitude of this saturable component (approximately 1–1.2 pmol·mg protein^−1^·10 min^−1^) was similar in both adipocytes and pre-adipocytes, although total uptake was about 50% higher in pre-adipocytes. An excess of the LNAA leucine (10 mM), inhibited over 80% of saturable T_3_ uptake (Figure 1). Pre-incubation of 3T3-L1 adipocytes in transport buffer containing 2 mM leucine for 15 minutes (followed by a rapid wash in PBS) resulted in a minor *trans* stimulatory increase in saturable 50 nM L-[^125^I]-T_3_ uptake (by 20 ± 8.5%, *n* = 3), although this increase did not reach statistical significance.

### 3.2. Phenylalanine Uptake

**Figure 2:**
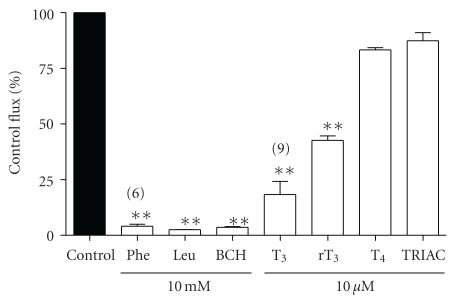
Effect of amino acids and iodothyronines on uptake of phenylalanine into 3T3-L1 adipocytes. [^3^H]phenylalanine uptake was measured at 5 *μ*M over 3 minutes in the absence (control) or presence of 10 *μ*M iodothyronine or 10 mM amino acid in the uptake buffer. Values are expressed ± SEM of 3 measurements (except where indicated) as % of the control flux. **P < .01; value significantly different from control using Dunnett's Multiple Comparison Test.

**Figure 3:**
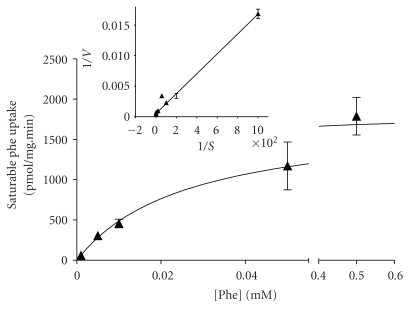
Saturable phenylalanine uptake into 3T3-L1 adipocytes. Uptake of [^3^H]phenylalanine over 3 minutes at 5 different phenylalanine concentrations as indicated (between 1 *μ*M and 500 *μ*M) was measured. Values are shown ± SEM for 3 measurements in a representative experiment and are expressed with the nonsaturable component of uptake removed. Figure inset shows a Lineweaver-Burk plot relating [^3^H]-phenylalanine uptake (corrected for nonsaturable component) against external phenylalanine concentration. The overall data provide estimated *K*_*m*_ of 31 *μ*M and *V*_max_ of 2.6 ± 0.5 nmol·mg protein^−1^·min^−1^ (mean ±sem, *n* = 6).

**Figure 4:**
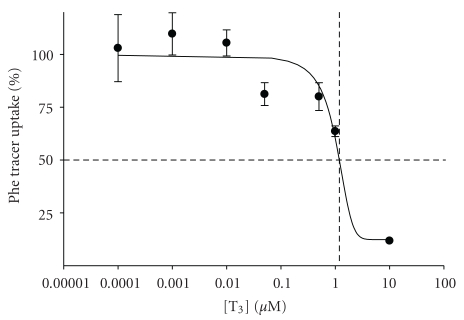
Concentration dependence of the inhibitory effect of tri-iodothyronine on phenylalanine uptake into 3T3-L1 adipocytes. Uptake of [^3^H]phenylalanine was measured at 5 *μ*M over 3 minutes in the presence of 7 different T_3_ concentrations as indicated (between 100 pM and 10 *μ*M). Values are expressed as % of control phenylalanine uptake (measured in the absence of T_3_). The dotted lines indicate that 50% inhibition is achieved with 1.2 *μ*M T_3_, estimated as the *K*_*i*_ for T_3_ inhibition of phenylalanine uptake in these cells.

The uptake of 5 *μ*M phenylalanine into 3T3-L1 cells (0.465 ± 0.065 nmol·mg protein^−1^·min^−1^, *n* = 12) was saturable, being reduced more than 95% by addition of excess (10 mM) unlabelled phenylalanine, leucine or BCH (2-aminobicyclo-(2,2,1)-heptane-2-carboxylic acid; a synthetic System L-type amino acid transport inhibitor) (Figure 2). 5 *μ*M phenylalanine uptake was also substantially inhibited by T_3_ and rT_3_ (82% and 57% inhibition, resp.) at 10 *μ*M, although inhibition by T_4_ (17%) and triiodothyroacetic acid (TRIAC; a T_3_ analogue lacking the *α*-amino grouping) did not achieve statistical significance at this concentration (Figure 2). Saturable phenylalanine uptake into 3T3-L1 cells had a *K*_*m*_ of 31 *μ*M and a *V*_max_ of 2.6 ± 0.5 nmol·mg protein^−1^·min^−1^ (mean ± sem, *n* = 6; see Figure 3). The inhibitory effect of T_3_ on phenylalanine uptake also appeared to be competitive, with a *K*_*i*_ of the order of 1-2 *μ*M (see Figure 4).

### 3.3. Phenylalanine Efflux

**Figure 5:**
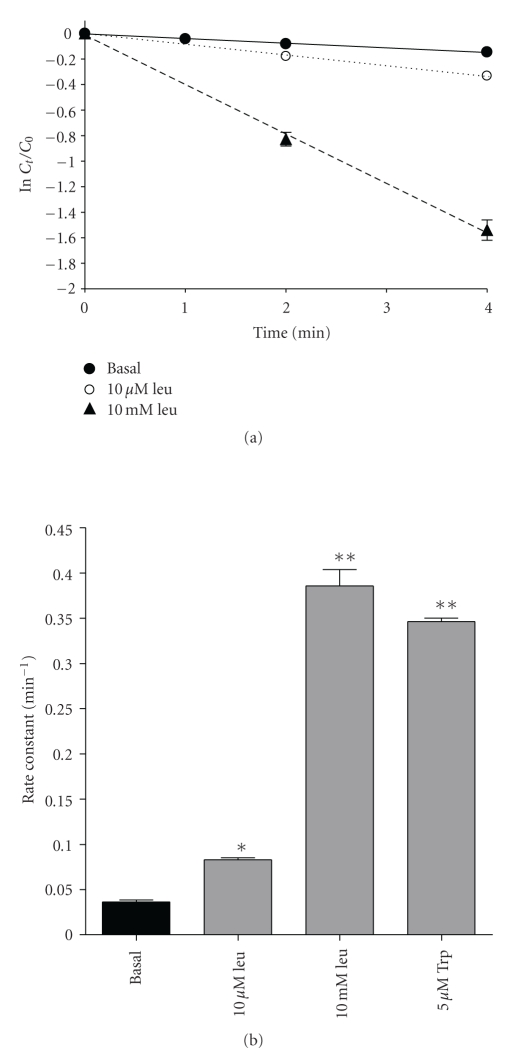
Phenylalanine efflux from 3T3-L1 adipocytes. (a) Time course of phenylalanine efflux showing the *trans* stimulatory effect of leucine. The cumulative release of [^3^H]phenylalanine from cells preloaded with the tracer was measured in the presence or absence of extracellular leucine over the time period indicated. Values are shown ± SEM for 6–18 measurements (smaller error bars are obscured by symbols). *C*_0_ represents the cellular radioactivity at the start of the experiment and *C*_*t*_ is that at time *t*, as calculated from the sum of cumulative radiotracer appearance in the medium and residual cellular radioactivity. The rate constant for efflux was taken as the gradient of the line of best fit. (b) Effects of external amino acids on rate constant for phenylalanine efflux. Bars indicate rate constants calculated as described in (a) and error bars represent SEM for gradient of the line of best fit. *P < .05, **P < .01; value significantly different from basal value.

**Figure 6:**
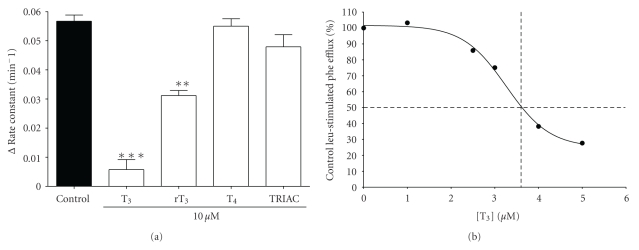
Triiodothyronine inhibits the *trans* stimulatory effect of leucine on phenylalanine efflux from 3T3-L1 adipocytes. (a) Effect of iodothyronines on leucine *trans* stimulation of phenylalanine efflux. Release of preloaded [^3^H]phenylalanine tracer from cells was measured in the presence or absence of 10 *μ*M leucine ± either T_3_, T_4_, rT_3_ or TRIAC (each at 10 *μ*M). The rate constants for efflux were calculated as described for Figure 4 and the change in rate constant from control (Δ rate constant) was calculated by subtracting the control efflux (without leucine) from that in the presence of leucine ± iodothyronine/TRIAC. Values are shown ± SEM for 12–18 measurements. *P < .05, **P < .01; value significantly different from control (leucine only) value. (b) Concentration dependence of T_3_ inhibition of leucine *trans* stimulated phenylalanine efflux. Release of preloaded [^3^H]phenylalanine tracer from cells was measured in the presence of 10 *μ*M leucine ± 5 different T_3_ concentrations (between 1 and 5 *μ*M) as indicated. The rate constants for efflux were calculated as described for Figure 4 and then as the percentage of efflux in the presence of leucine alone. Values are shown ± SEM for 6 measurements (data points obscure error bars). The dotted lines indicate that 50% inhibition of 10 *μ*M leucine *trans* stimulated phenylalanine efflux is achieved with 3.2 *μ*M T_3_.

Semilogarithmic plots of the cumulative loss of preloaded [^3^H]phenylalanine tracer from 3T3-L1 cells showed that the initial efflux was described by a single rate constant (see Figure 5(a)), indicating that phenylalanine was released largely from a single, freely exchangeable intracellular pool. Efflux of pre-loaded L-[^3^H]phenylalanine from 3T3-L1 adipocytes was *trans* stimulated in a concentration-dependent manner by external leucine (Figure 5(a)). Leucine and tryptophan (both System L substrates) were able to generate over 20 times stimulation of basal phenylalanine efflux (Figure 5(b)). T_3_ is known to be a substrate for System L1 amino acid transport, so we investigated the ability of T_3_ to *trans* stimulate phenylalanine efflux. T_3_ was unable to significantly *trans* stimulate L-[^3^H]phenylalanine efflux at 10 *μ*M (a concentration approaching the limit of T_3_ solubility in culture medium), although it did inhibit the *trans* stimulatory effect of 10 *μ*M leucine (see Figure 6(a)). rT_3_ also inhibited the *trans* stimulatory effect of 10 *μ*M leucine, although T_4_ did not to any significant degree (Figure 6(a)). The T_3_ analogue TRIAC (which does not interact with System L1) had no effect on either basal or leucine-stimulated phenylalanine efflux. The effect of T_3_ upon leucine *trans* stimulated phenylalanine efflux was found to be concentration dependent (see Figure 6(b)) with 3.2 *μ*M T_3_ able to inhibit 50% of 10 *μ*M leucine *trans* stimulated phenylalanine efflux, demonstrating the relatively high affinity of T_3_ for this interaction.

### 3.4. Transporter mRNA Expression

**Figure 7:**
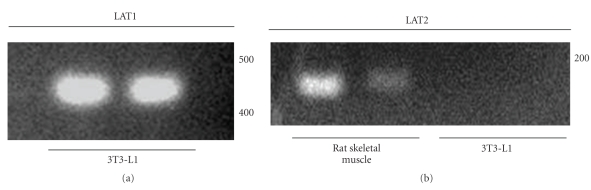
LAT1, a catalytic subunit for System L1 transport, is expressed in 3T3-L1 adipocytes at the mRNA level but LAT2 is not. Reverse transcriptase PCR (RT-PCR) using gene-specific primers designed towards either LAT1 or LAT2 was performed with template cDNA produced from RNA extracted from 3T3-L1 adipocytes. Samples were separated using 2% agarose alongside a 1kb molecular weight marker (Roche Applied Science). (a) LAT1 mRNA is expressed in 3T3-L1 adipocytes. Following agarose gel electrophoresis, a band of 150 bp was observed corresponding to the expected PCR amplicon using LAT1-specific primers as detailed in the text. (b) LAT2 mRNA is not expressed in 3T3-L1 adipocytes. Following agarose gel electrophoresis, no PCR amplicon was detected when 3T3-L1-derived cDNA was used as a template. However, PCR using LAT2-specfic primers and cDNA derived from rat skeletal muscle did amplify a band of the expected size (465 bp), as shown, which demonstrates the functionality of the primers used.

We detected mRNA for LAT1, but not LAT2 (the alternative catalytic subunit producing System L1-type amino acid transport) in 3T3-L1 cells using RT-PCR (see Figure 7).

## 4. Discussion

3T3-L1 cells in culture are used extensively as an *in vitro* model system for studying white adipose tissue [21] and the present results demonstrate that saturable uptake of the thyroid hormone L-T_3_ in both pre-adipocytes and terminally differentiated 3T3-L1 adipocytes occurs largely by a mechanism inhibited by the LNAA leucine, a substrate of the multifunctional System L1 transport system shared by LNAA and iodothyronines (see [6] for review). Saturable uptake processes appear to be the predominant routes of cellular TH entry for genomic signaling *via* thyroid receptors in animal cells [7]. There appears to be little change in functional expression of saturable T_3_ transport during adipocyte differentiation (at least for 3T3-L1 cells), which is perhaps unsurprising given that T_3_ is important for the differentiation process itself as well as the control of mature adipocyte functions [10]. The nonsaturable “uptake” of T_3_ into adipocytes includes surface binding (presumably to secreted lipids and lipoproteins) and partitioning into the lipid bilayer as well as passive diffusion into the cytosol [15]. Transport of phenylalanine (a representative LNAA substrate for Systems L1, L2 and T) in 3T3-L1 adipocytes conforms almost exclusively with “classical” System L1-type function as mediated by LAT1 [23], in that (i) phenylalanine influx has a *K*_*m*_ in the low micromolar range (31 *μ*M), (ii) it is inhibited by BCH and (iii) phenylalanine efflux is markedly *trans* stimulated in a concentration-dependent manner by both leucine and tryptophan (consistent with the obligatory exchange mechanism of System L1 [24]). These features are clearly distinct from those of other likely contributors to phenylalanine transport, specifically because (i) System L2 (LAT3/LAT4) does not recognize tryptophan as a substrate [25, 26], (ii) System T (MCT10, originally named TAT1) does not recognize leucine as a substrate [27, 28], and (iii) LAT3, LAT4, and MCT10 all utilise facilitative diffusion rather than an exchange mechanism [25–27]. Phenylalanine uptake by 3T3-L1 cells was competitively inhibited by T_3_ (*K*_*i*_ of 1-2 *μ*M) and rT_3_ in a manner consistent with expected substrate interactions for the LAT1 System L1 transporter [29–32]. This strong competitive interaction between iodothyronines and LNAA for transport by System L1 in 3T3-L1 mouse adipocytes is consistent with our previous studies using isolated white adipocytes from rats [15], although the relatively-weak inhibitory effects of T_4_ in the present study may reflect minor species-specific differences in the relative affinity of different TH for LAT1. The *V*_max_ for phenylalanine transport in 3T3-L1 cells (2.6 nmol·mg protein^−1^·min^−1^) is broadly equivalent to 50 pmol·10^5^ cells^−1^·min^−1^, a value of the same order of magnitude as that measured for System L1 transport in rat adipocytes [15].

Collectively, our results indicate that the Na^+^-independent System L1 transporter contributes substantially to iodothyronine and LNAA transport in 3T3-L1 adipocytes and thus for delivery of TH from the plasma to the cytosol, where ultimately they reach the cell nucleus. System L1 has been shown to be an important effector of T_3_ transport in tissues including placenta and brain as well as adipose ([6] for review). The LAT1 and LAT2 genes encode for alternative catalytic protein subunits of System L1 and these function as heterodimers with 4F2hc/CD98 (see [23] for review). LAT1 and LAT2 both mediate uptake of iodothyronines as well as LNAA [30, 31] but while both are expressed in rat adipocytes [16], it appears that only LAT1 is expressed at the mRNA level in 3T3-L1 cells. Our transport studies also indicate the predominant functional expression of LAT1 in 3T3-L1 cells. The *K*_*m*_ values obtained for iodothyronine transport by System L1 are substantially lower than those measured for LNAA transport, which help T_3_ especially to compete effectively with amino acids for transport under physiological conditions (see [6] for review). High-affinity receptors for both T_4_ and T_3_ have been identified associated with the plasma membrane of adipocytes [33] and these may also facilitate preferential iodothyronine uptake [15]. To our knowledge, T_3_ is the most potent natural inhibitor of System L1 in the mammalian body (and easily surpasses the widely-used synthetic inhibitor BCH in terms of *K*_*i*_, if not specificity), although Brasilicardin compounds isolated from bacteria have recently been shown to inhibit with nanomolar potency [34]. Given the therapeutic potential of System L1 inhibitors as immunosuppressants and anticancer drugs [34, 35], it is tempting to suggest that the iodothyronine skeleton might be used as a template for improved design of highly specific, non-TH active, System L1 inhibitors. Reverse T_3 _but not TRIAC (a T_3_ analog lacking an amino acid moiety which acts as a potent thyroid receptor agonist) interacts with System L1 in adipocytes, demonstrating structural specificity of System L1 which is clearly distinct from that of nuclear thyroid hormone receptors. TRIAC selectively stimulates the metabolic rate of adipose tissue *in vivo*, a feature considered as a potential tool to increase whole body energy metabolism [13]. It appears from our results that TRIAC enters adipocytes by a different mechanism to T_3_, which may help explain why TRIAC administered to rats accumulates in white adipose tissue even when coadministered with high T_3_ doses [13].

The bulky T_3_ molecule has a much reduced *V*_max_ for transport by System L1 compared to LNAA [30], such that its specific interactions with System L1 transport may competitively inhibit not only influx but efflux of substrate LNAA by retarding conformational changes of the transport cycle (note the inhibitory action of T_3_ on leucine-induced *trans* stimulation of phenylalanine efflux in 3T3-L1 cells). Nevertheless, the obligatory exchange mechanism of the System L1 transporter should allow limited LNAA–iodothyronine or T_4_-T_3_/rT_3_ exchanges across the adipocyte plasma membrane and we indeed observed a minor *trans* stimulation of saturable L-T_3_ uptake in 3T3-L1 adipocytes preincubated with leucine. It is noteworthy in this respect that adipose tissue *in vivo* releases small quantities of LNAA [36]. White adipose tissue depots have the capacity to deiodinate iodothyronines (especially rT_3_) [37, 38], thus it is conceivable that regulation of iodothyronine turnover in fat cells through this pathway would contribute significantly to modulation of whole body T_4_-rT_3_ metabolism, perhaps particularly in obese individuals. In this respect, downregulation of iodothyronine uptake by System L1 in adipocytes in the hypothyroid state, as reported previously in adipocytes from hypothyroid rats [15], would tend to reduce the sensitivity of adipose tissue to hormonal activation but might help conserve hormone and iodine availability to other tissues by reducing their degradation in adipose tissue. Physiological variations in plasma LNAA concentrations, as occurs during natural feeding cycles, may impact TH transport and action in adipose (and other tissues where System L1 is the predominant TH transporter).

## Abbreviations

BCAA: Branched chain amino acids
BCH: 2-amino [2,2,1] heptane–2-carboxylic acid
T_3_: L-triiodothyronine
T_4_: L-thyroxine
rT_3_: Reverse L-triiodothyronine
TH: thyroid hormone
TRIAC: triiodothyroacetic acid.
